# *FUT2* and *FUT3*-specific normalization of DUPAN-2 and carbohydrate antigen 19-9 in preoperative therapy for pancreatic cancer: multicentre retrospective study (GEMINI-PC-01)

**DOI:** 10.1093/bjs/znaf049

**Published:** 2025-04-29

**Authors:** Haruyoshi Tanaka, Ayano Sakai, Masaya Suenaga, Masamichi Hayashi, Tomohisa Otsu, Nobuhiko Nakagawa, Keisuke Kurimoto, Mina Fukasawa, Kazuto Shibuya, Nobuyuki Watanabe, Masaki Sunagawa, Junpei Yamaguchi, Takashi Mizuno, Toshio Kokuryo, Hideki Takami, Tomoki Ebata, Tsutomu Fujii, Yasuhiro Kodera

**Affiliations:** Department of Surgery, Nagoya University Hospital, Nagoya, Japan; Department of Surgery and Science, Faculty of Medicine, Academic Assembly, University of Toyama, Toyama, Japan; Department of Surgery and Science, Faculty of Medicine, Academic Assembly, University of Toyama, Toyama, Japan; Department of Surgery, NHO Nagoya Medical Centre, Nagoya, Japan; Department of Surgery, Nagoya University Hospital, Nagoya, Japan; Department of Surgery, Nagoya University Hospital, Nagoya, Japan; Department of Surgery, Nagoya University Hospital, Nagoya, Japan; Department of Surgery, Nagoya University Hospital, Nagoya, Japan; Department of Surgery and Science, Faculty of Medicine, Academic Assembly, University of Toyama, Toyama, Japan; Department of Surgery and Science, Faculty of Medicine, Academic Assembly, University of Toyama, Toyama, Japan; Department of Surgery, Nagoya University Hospital, Nagoya, Japan; Department of Surgery, Nagoya University Hospital, Nagoya, Japan; Department of Surgery, Nagoya University Hospital, Nagoya, Japan; Department of Surgery, Nagoya University Hospital, Nagoya, Japan; Department of Surgery, Nagoya University Hospital, Nagoya, Japan; Department of Surgery, Nagoya University Hospital, Nagoya, Japan; Department of Surgery, Nagoya University Hospital, Nagoya, Japan; Department of Surgery and Science, Faculty of Medicine, Academic Assembly, University of Toyama, Toyama, Japan; Department of Surgery, Nagoya University Hospital, Nagoya, Japan

*Dear Editor*

Pancreatic cancer is one of the most challenging cancers to treat^1^. Some patients with unresectable disease at diagnosis may achieve a remarkable response by multimodal therapy and undergo subsequent surgery (so-called ‘conversion surgery’). However, determining the appropriate indications for conversion surgery often presents a formidable challenge^1–3^. In addition to carbohydrate antigen 19-9 (CA19-9), DUPAN-2, and *FUT2* and *FUT3,* or *FUT2/3* status (*FUT2*-null, *FUT*-intact, and *FUT3*-null) are emerging biomarkers for pancreatic cancer^4,5^. However, studies on integrating these biomarkers during preoperative therapy are lacking. The aim of this study was to examine changes in CA19-9 and DUPAN-2 levels according to *FUT2/3* status during preoperative therapy and to test whether the determination of genotype-specific tumour marker normalization can enhance the usefulness of these markers when considering surgery.

This study was the retrospective part of a multicentre observational study (GEMINI-PC-01/02). A total of 347 patients were enrolled who underwent pancreatectomy with curative intent after preoperative treatment for pancreatic cancer. After excluding two patients because of their final pathology and four patients because of mis-genotyping using a TaqMan-PCR panel (*supplementary methods*), which was designed considering the variant allele frequency in Japan (*Table S1*), data for 341 patients were used in the analyses.

The patients’ characteristics stratified by resectability are shown in *Table S2* (see *Table S3* for metastatic disease). The distributions of CA19-9 and DUPAN-2 were more distinct for *FUT2/3* status compared with resectability (*Fig. 1a*; see *Fig. S1a* for the sub-subset analysis). Overall survival did not differ greatly by resectability or *FUT2/3* status (*Fig. 1b* and *Fig. 1c* respectively; see *Fig. S1b* for the sub-subset analysis). However, a significant prognostic difference across *FUT2/3* status was observed in the unresectable subset (*Fig. 1d*). Therefore, the authors focused on the unresectable subset (*Table S4*) to develop a novel prognostic model, called the tumour marker gene model (TMGM). Using genotype-specific cut-offs, the authors categorized patients whose marker levels were lower than the cut-offs as TMGM-low and the remaining patients as TMGM-high (*Fig. 1e*; see *Fig. S2* and the *supplementary results* for background information regarding the definitions). The TMGM successfully stratified patient prognosis compared with the compound single cut-off model (*Fig. 1f*; see *Fig. S3* for the other models and *Fig. S4* for the sub-subset analyses). The Cox proportional hazards analysis identified TMGM-high as an independent prognostic factor (*Table 1*).

**Fig. 1:**
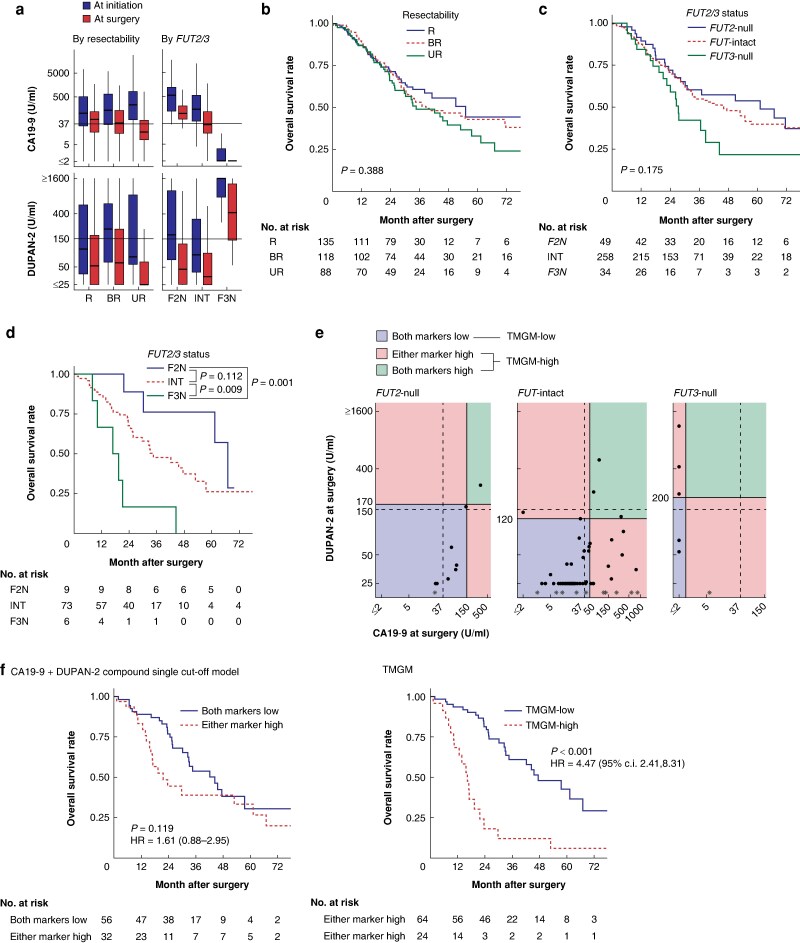
*FUT2* and *FUT3* status greatly affects carbohydrate antigen 19-9 and DUPAN-2 expression and might have impacted their prognoses, potentially due to the baseline changes of these markers. The Tumor Marker Gene Model was develped to demonstrated good prognostic predicting ability **a** CA19-9 and DUPAN-2 levels according to resectability (left-hand panels) and *FUT2/3* status (right-hand panels). The *y*-axis is presented on a log-10 scale. **b** Overall survival curves according to resectability. **c** Overall survival curves according to *FUT2/3* status. **d** Overall survival curves for the unresectable subset according to *FUT2/3* status. Differences in survival were tested using the log rank or pairwise log rank test for post-hoc analysis. **e** Schema of the TMGM. The schema shows the distribution of CA19-9 and DUPAN-2 levels at surgery in the unresectable subset. The continuous lines indicate genotype-specific cut-offs and the broken lines indicate uniform upper limits (refer to the *Supplementary material* for the background information). An asterisk indicates that data were not available for the DUPAN-2 level. **f** Overall survival curves stratified by the compound single cut-off model (left-hand panel) and the TMGM (right-hand panel). CA19-9, carbohydrate antigen 19-9; R, resectable; BR, borderline resectable; UR, unresectable; F2N, *FUT2*-null; INT, *FUT*-intact; F3N, *FUT3*-null; TMGM, tumour marker gene model.

**Table 1:** Cox proportional hazards analysis for overall survival after surgery

Variables	Terms	*n*	Univariable		Multivariable	
			HR (95% c.i.)	*P*	HR (95% c.i.)	*P*
Sex	Male	48	1.00 (0.55,1.79)	0.99		
Age	>75 years	13	1.18 (0.53,2.66)	0.69		
Tumour location	Head	65	1.03 (0.53,2.01)	0.93		
Metastasis, at diagnosis	Yes	18	1.72 (0.88,3.34)	0.11		
New era chemotherapy*	Yes	66	0.76 (0.40,1.44)	0.40		
Treatment duration	>8 months	49	0.82 (0.45,1.47)	0.50		
Tumour diameter, at diagnosis	>40 mm	22	1.74 (0.95,3.18)	0.071		
Tumour diameter, at surgery	>20 mm	42	1.85 (1.02,3.34)	0.043†	1.88 (1.03,3.42)	0.040†
RECIST classification	PR or more	47	0.80 (0.42,1.51)	0.48		
Tumour markers, single cut-off model‡	High	32	1.61 (0.88,2.95)	0.12		
Tumour markers, TMGM	High	24	4.47 (2.41,8.31)	<0.001†	4.56 (2.44,8.59)	<0.001†
Evans classification	IIb or more	48	0.76 (0.41,1.42)	0.39		
Residual tumour (*versus* R0)	R1 or R2	18	1.35 (0.65,2.82)	0.42		

*New era chemotherapy includes gemcitabine plus nab-paclitaxel and fluorouracil, leucovorin, irinotecan, plus oxaliplatin (FOLFIRINOX). †Statistically significant. ‡CA19-9 >37 U/ml or DUPAN-2 >150 U/ml. RECIST, response evaluation criteria in solid tumours; TMGM, tumour marker gene model.

Next, the TMGM was investigated further to verify its usefulness in practical applications. First, the TMGM and the DUPAN-2 single cut-off model were compared, which appears to be a good standalone predictor^5^. The TMGM was superior to the DUPAN-2 single cut-off model on the basis of Harrel’s concordance index (0.674 *versus* 0.569 respectively). Second, it was observed how the TMGM converged to TMGM-low, suggesting the best time to perform surgery. Data for 26 representative patients who had undergone preoperative therapy for 8–12 months were selected. The chronological changes in the two markers for each genotype showed more genotype-specific CA19-9 normalization compared with DUPAN-2 (*Fig. S5*).

In summary, *FUT2/3* status greatly affected CA19-9 and DUPAN-2 levels during preoperative therapy and the TMGM was successfully developed to stratify patient prognosis better than conventional single cut-off models. Unexpectedly, *FUT3*-null demonstrated a poor prognosis in the unresectable subset. Currently, the authors do not consider this finding a result of tumour biology but, instead, misinterpretation of tumour marker normalization, which is a pivotal surgical indication. Therefore, the authors decided to develop the TMGM with a focus on unresectable diseases. Physicians may have overestimated tumour marker normalization in this study because of deficient CA19-9 levels in *FUT3*-null patients. Some of them may benefit from avoiding surgery and continuing induction therapy. In contrast, potentially more *FUT2*-null patients could have undergone conversion surgery, even if they had relatively high CA19-9 and DUPAN-2 levels. Furthermore, conversion surgery for metastatic disease is beyond the scope of current guidelines. However, personalized treatment strategies can encompass conversion surgery as a possible option in select cases.

The present study has certain limitations associated with its retrospective design. In particular, the study cohort included only patients who underwent surgery and the indications for surgery after preoperative therapy were not predefined. Second, the sample size of the unresectable subset was insufficient to consider the prognostic model robust. Prospective and/or large cohort studies, preferably registered at an early stage of induction therapy, are needed to validate the results of the present study.

## Supplementary Material

znaf049_Supplementary_Data

## Data Availability

The data generated in this study are available from the corresponding author upon reasonable request.
