# Supplementary material for: FUT2 and FUT3-specific normalization of DUPAN-2 and carbohydrate antigen 19-9 in preoperative therapy for pancreatic cancer: multicentre retrospective study (GEMINI-PC-01)
Source: Br J Surg. 2025 Apr 29;112(4):znaf049. doi: 10.1093/bjs/znaf049 (PMC12037208; doi:10.1093/bjs/znaf049)
Supplement: znaf049_Supplementary_Data [file znaf049_supplementary_data.docx]

**Title: FUT2/3-specific normalization of DUPAN-2 and CA19-9 in preoperative therapy for pancreatic cancer: a multi-center retrospective study (GEMINI-PC-01)**

Authors: Haruyoshi Tanaka, MD, PhD^1,2^, Ayano Sakai (contributed equally), MD^2^, Masaya Suenaga, MD, PhD^3^, Masamichi Hayashi, MD, PhD, FACS^1^, Tomohisa Otsu, MD^1^, Nobuhiko Nakagawa, MD, PhD^1^, Keisuke Kurimoto, MD, PhD^1^, Mina Fukasawa, MD^2^, Kazuto Shibuya, MD, PhD^2^, Nobuyuki Watanabe, MD, PhD^1^, Masaki Sunagawa, MD, PhD^1^, Junpei Yamaguchi, MD, PhD^1^, Takashi Mizuno, MD, PhD^1^, Toshio Kokuryo, MD, PhD^1^, Hideki Takami, MD, PhD^1^, Tomoki Ebata, MD, PhD^1^, Tsutomu Fujii, MD, PhD, FACS^2^, Yasuhiro Kodera, MD, PhD, FACS^1^

**Affiliations:**

^1^ Department of Surgery, Nagoya University Graduate School of Medicine, Nagoya, Japan

^2^ Department of Surgery and Science, Faculty of Medicine, Academic Assembly, University of Toyama, Toyama, Japan

^3^ Department of Surgery, NHO Nagoya Medical Center, Nagoya, Japan

**Address all correspondence to:**

Haruyoshi Tanaka, Department of Surgery, Nagoya University Graduate School of Medicine,

466-8550, Tsurumai-cho 65, Nagoya, Japan; Telephone, +52-744-2249; fax, +52-744-2252;

E-mail, tanaka.haruyoshi.f1@f.mail.nagoya-u.ac.jp

**ORCID ID**, 0000-0003-0079-8422; **X (formerly, Twitter),** @haruween1

**Supplementary Materials - Index**

| **Supplementary Methods** |  |
| --- | --- |
| Study population and design | *page 3* |
| Principal therapeutic policy for pancreatic cancer | *page 4* |
| Determination of FUT2/3 genotypes | *page 4* |
| Ethical issues | *page 5* |
| Statistical analysis | *page 6* |
| **Supplementary Results** |  |
| Characteristics of the study population | *page 6* |
| Survival analysis and tumor marker gene model | *page 7* |
| **Supplementary Appendices** |  |
| Authors’ contributions | *page 9* |
| **Supplementary Figures and Tables** |  |
| Supplementary Figure 1 (Fig. S1) | *page 11* |
| Supplementary Figure 2 (Fig. S2) | *page 12* |
| Supplementary Figure 3 (Fig. S3) | *page 13* |
| Supplementary Figure 4 (Fig. S4) | *page 14* |
| Supplementary Figure 5 (Fig. S5) | *page 15* |
| Supplementary Table 1 (Table S1) | *page 16* |
| Supplementary Table 2 (Table S2) | *page 17* |
| Supplementary Table 3 (Table S3) | *page 19* |
| Supplementary Table 4 (Table S4) | *page 20* |
| **References** | *page 21* |

**Supplementary Methods**

**Study population and design**

The present study was the retrospective part of a multicenter observational cohort study (GEMINI-PC-01/02, jRCT1040230005). The eligibility criteria are described at https://jrct.niph.go.jp/latest-detail/jRCT1040230005. Briefly, we collected data for 347 patients who underwent pancreatectomy with curative intent after preoperative treatment for pancreatic cancer between January 2012 and December 2021 in three institutions located in central Japan. Lymphocytes, frozen tissue, or formalin-fixed paraffin-embedded (FFPE) tissues without cancer (e.g., duodenum or spleen) were collected. After excluding two patients owing to their final pathology (carcinoma of the ampulla of Vater and neuroendocrine tumor, one each), 345 patients were genotyped. Among those who were tested, 4 patients whose genotyping failed were excluded, and data for 341 individuals were included in the analyses. Owing to the retrospective and exploratory nature of this study, randomization of the subjects or formal power calculation was not required.

Clinical variables, including sex, age, and all other possible surgical and oncological demographics, were collected from institutional electronic medical records. In particular, resectability was assessed by radiologists using dynamic computed tomography (CT) at the initial presentation in accordance with the latest versions of the National Comprehensive Cancer Network guidelines (https://www.nccn.org/guidelines/category_1). In this study, locally advanced and metastatic diseases were defined as unresectable.

Carbohydrate 19-9 (CA19-9) and DUPAN-2 levels at the initiation of preoperative therapy were recorded after biliary drainage for obstructive jaundice, if present. CA19-9 levels were measured in the clinical laboratory of each institution, and there were no missing data at the time of surgery. DUPAN-2 levels were measured by SRL, Inc. (Tokyo, Japan), which uses a commercially available DUPAN-2 enzyme immunoassay kit (Determiner-DUPAN-2 N; Minaris Medical Co., Ltd., Tokyo, Japan). The minimum and maximum ranges of the calibration curve of the kit were 25 U/ml and 1600 U/ml, respectively. DUPAN-2 levels at surgery were missing in 41 patients.

**Principal therapeutic policy for pancreatic cancer**

Our policy for preoperative therapy was as follows: short-term neoadjuvant chemotherapy for resectable disease^1^ and intensive neoadjuvant chemotherapy with or without adjunctive radiotherapy for borderline resectable disease; surgical eradication was planned unless there was progressive disease^2^. For unresectable disease, conversion surgery was considered when the disease was well controlled, with partial response or long stable disease on radiological tests (generally > 6 months) and regarding tumor markers, as described previously^3^. In particular, for locally advanced disease, surgical exploration and rapid pathological examination using frozen sections were performed as needed at the time of conversion surgery to determine whether radical resection was feasible. For metastatic disease, we diagnosed metastases using multiple modalities, such as CT, gadolinium-ethoxybenzyl-diethylenetriamine pentaacetic acid-enhanced magnetic resonance imaging, positron emission tomography-CT, and staging laparoscopy, depending on the metastatic probability^4^. If the metastatic foci achieved a remarkable response to induction therapy, staging laparoscopy was performed to evaluate peritoneal cytology and perform excisional biopsy of the metastatic foci. If pathological testing revealed no residual cancer, conversion surgery was performed. Alternatively, if the testing revealed the foci residual cancer and all of those were macroscopically eradicated by that excisional biopsy, the same regimen was continued for several months followed by conversion surgery, unless progressive disease occured^5, 6^.

**Determination of FUT2/3 genotypes**

Genomic DNA from normal tissue frozen or lymphocytes was extracted using a QIAamp DNA Mini Kit (Qiagen, Hilden, Germany), and that from FFPE extracted using QIAamp DNA FFPE Tissue Kit (Qiagen, Germany) as described previously^7, 8^. TaqMan polymerase chain reaction testing was used to genotype FUT2/3. Three TaqMan genotyping assays were performed to test the three loci, considering variant allele frequencies in the Japanese population, in accordance with previous reports ^9^ and TOGO-VAR (https://togovar.biosciencedbc.jp/) (**Table S1**). Two of these were predesigned (Assay ID, C___8832449_10 for FUT2 gene A385T, and C___8832450_10 for FUT2 gene C357T; Thermo Scientific, Waltham, MA, USA). The other assay for FUT3 gene T59G was designed for this study. The oligonucleotide sequences of the primers and probes were as follows: Forward primer, ATGGCGCCGCTGTCT; Reverse primer, GCAGGTAGGAGAAGAAACACACA; probe for wild-type, VIC-CTGAAATAGCAGTGCGGC-NFQ-MGB; and probe for the variant, FAM-CTGAAATAGCCGTGCGGC-NFQ-MGB. The TaqMan Genotyping Master Mix (Thermo Fisher Scientific) was used as the reaction mixture. Polymerase chain reaction cycling conditions were as follows: one cycle of polymerase activation at 95°C for 10 min, followed by 40 cycles of denaturation at 95°C for 15 s, and annealing and extension at 60°C (58°C for FUT3 T59G) for 1 min. Owing to the nature of discrete or qualitative variables, the genotyping test was performed in a single assay, unless the result was equivocal or no amplification was observed. To mitigate bias, patient information, including prognosis, was blinded to the genotyping experimenter until the test was completed. Classification into three groups in accordance with FUT2 and FUT3 variants was performed as follows: FUT3-null was defined as the presence of a homozygous variant of T59G regardless of FUT2 gene status; FUT2-null was defined as the presence of a homozygous variant at both FUT2 A385T and FUT2 C357T; and the others were defined as FUT-intact^7, 10, 11^.

**Ethical issues**

This study conformed to the ethical guidelines of the World Medical Association Declaration of Helsinki Ethical Principles for Medical Research Involving Human Subjects. Written informed consent was obtained from all of the patients for the use of their blood samples. Patients whose FFPE samples were used were deemed to have consented to the present study with an opt-out policy, and the need to obtain in-person consent was waived. The procedures were approved by the Institutional Review Board of Nagoya University, Japan (approval ID: 2021-0385). We were encouraged to report the present study in accordance with the Strengthening the Reporting of Observational Studies in Epidemiology (STROBE) Statement (https://www.strobe-statement.org/).

**Statistical analysis**

The significance of the difference between the variables was assessed using Student’s t-test or the Mann–Whitney test between two groups, and the paired t-test and Kruskal–Wallis test between three or more groups, depending on their distribution. Data are presented as mean ± standard division or median with interquartile range. Fisher’s exact test was used to analyze the categorical data of the two groups. Overall survival (OS) was defined as the interval between the date of surgery and the date of the last follow-up or death. Survival curves were generated using the Kaplan‒Meier method, and their differences were evaluated by the log-rank test. The pairwise log-rank test adjusted by the Benjamini‒Hochberg method was used to compare three groups. Survival analysis, including a Cox proportional hazards regression model and calculation of the concordance index^12^, was performed using the survival package in R (version 3.5.3; Therneau, 2020; https://CRAN.R-project.org/package=survival). To develop genotype-specific prognostic models, CA19-9 and DUPAN-2 cutoffs for each FUT2/3 genotype were set using the minimum *P*-value method^13, 14^. All statistical analyses were performed using R software (The R Foundation for Statistical Computing, Vienna, Austria) version 4.2.3.

**Supplementary Results**

**Characteristics of the study population**

Genotyping was successful in 341 of 345 patients. The distribution of the FUT2/3 genotype was not significantly different among the resectability groups (*P* = 0.421), which was comparable to results in a previous study^9^. The patients’ characteristics stratified by resectability are shown in **Table S2**. The unresectable (UR) subset included 70 locally advanced (UR-LA) and 18 metastatic disease (UR-M) sub-subsets. Many variables were distinct on the basis of resectability: more radiologic and pathologic responses were observed, and more concomitant vascular resections were performed in advanced cases. One patient died postoperatively in this cohort, and morbidities were comparable across resectability categories. Patients with unresectable disease had a longer preoperative treatment duration than those with resectable and borderline resectable disease. **Table S3** shows the detailed characteristics of the patients with metastatic disease. Most primary lesions were resectable, and (cyto)pathological proof was obtained in most patients during multidisciplinary therapy. Owing to the invisibility of metastatic foci, these were sampled surgically to obtain proof of chemotherapeutic eradication in half of the patients.

Tumor markers were drastically distinct regarding treatment stages (at initiation of therapy and at operation), resectability, and especially, the FUT2/3 genotype. Generally, CA19-9 levels decreased after preoperative therapy. CA19-9 and DUPAN-2 levels in patients with unresectable disease were higher than those of other patients at initiation of therapy and appeared to converge at the lowest levels at surgery, presumably owing to a longer duration of induction therapy (**Fig. 1a** and **Fig. S1a** for the sub-subset analysis). The distributions of CA19-9 and DUPAN-2 levels were more distinct among the FUT2/3 genotype groups than among the resectability groups (**Fig. 1a**). In particular, the CA19-9 levels were generally low in the FUT3-null group.

**Survival analysis and tumor marker gene model**

The median OS for patients with resectable, borderline resectable, and unresectable disease was 55.0, 38.6, and 34.6 months, respectively (**Fig. 1b**). Although survival appeared to be better in the following order: R > BR > UR-LA > UR-M, there was no significant difference between the groups; median OS was 44.4 and 25.4 months for UR-LA and UR-M, respectively (**Fig. S1b**). Sub-subset analysis also showed no significant differences in survival by resectability. The median OS for the FUT2-null, FUT-intact, and FUT3-null groups was 61.3, 46.1, and 27.4 months, respectively (**Fig. 1c**). Subgroup analysis by resectability revealed a significant prognostic difference across genotypes only in the unresectable subset (median OS: 67.0, 34.5, and 18.1 months, respectively) (**Fig. 1d**). In this subset, the FUT3-null group had a significantly worse survival compared with the FUT2-null group, with a hazard ratio (HR) of 7.94 (95% confidence interval: 2.13–29.6) and FUT-intact group (HR = 3.38 [95% confidence interval: 1.40–8.18]).

Next, we examined the unresectable subset (n = 88) to identify genotype-specific optimal cutoffs for the two markers after preoperative therapy. The characteristics of these patients were similar among the FUT2/3 genotypes (**Table S4**) except for CA19-9 and DUPAN-2 (**Fig. 1e**). Using the minimum P-value method^13, 14^, the CA19-9 cutoffs were set to 150 U/ml in the FUT2-null group, 50 U/ml in the FUT-intact group, and 2 U/ml in the FUT3-null group, unless the CA19-9 level was ≤ 2 U/ml at both initiation and surgery; DUPAN-2 cutoffs were set to 170, 120, and 200 U/ml, respectively. Using the above FUT2/3-specific CA19-9 and DUPAN-2 cutoffs, termed the tumor marker gene model (TMGM), the unresectable cohort was classified into three groups: 64 patients had lower CA19-9 and DUPAN-2 levels (Both-low group); 12 patients had high CA19-9 or DUPAN-2 levels (Either-high group); and 12 patients had high CA19-9 and DUPAN-2 levels (Both-high group) (**Fig. 1e**). OS was better in the Both-low group compared with the Either-high and Both-high groups, with a median OS of 47.4, 19.4, and 16.2 months, respectively (**Fig. S2**). We then categorized the patients in the Both-low group into the TMGM-low group, and the other patients into the TMGM-high group (**Fig. 1e**). The unresectable subset was also categorized by single cutoffs: 37 U/ml for CA19-9, unless CA19-9 was ≤ 2 U/ml at both diagnosis and surgery, and 150 U/ml for DUPAN-2. Additionally, those whose CA19-9 and DUPAN-2 levels were lower than each single cutoff were categorized as the low group, and this model was termed the compound single cutoff model.

TMGM successfully stratified patient prognosis better than the compound single-cutoff model (**Fig. 1f**; *P* < 0.0001; HR = 4.47 [2.41–8.31] for TMGM; see **Fig. S3** for other single-cutoff models). TMGM also successfully stratified survival in both the locally advanced and metastatic sub-subsets (**Fig. S4**). The Cox proportional hazard model identified TMGM-high as an independent prognostic factor (**Table 1**).

**Supplementary Appendices**

**Author contributions**

Haruyoshi Tanaka (Conceptualization, Data curation (clinical and experimental), Formal analysis, Investigation, Methodology, Visualization, Writing—original draft), Ayano Sakai (Data curation (clinical and experimental), Formal analysis, Investigation, Methodology, Visualization, Writing—original draft), Masaya Suenaga (Conceptualization, Data curation (clinical and experimental), Investigation, Methodology, Writing—review & editing), Masamichi Hayashi (Conceptualization, Data curation (experimental), Methodology, Supervision, Writing—review & editing), Tomohisa Otsu (Data curation (clinical and experimental), Writing—review & editing), Nobuhiko Nakagawa, (Data curation (clinical), Writing—review & editing), Keisuke Kurimoto, (Data curation (clinical), Writing—review & editing), Mina Fukasawa, (Data curation (clinical), Writing—review & editing), Kazuto Shibuya (Data curation (clinical), Writing—review & editing), Nobuyuki Watanabe (Data curation (clinical), Writing—review & editing), Masaki Sunagawa (Data curation (clinical), Writing—review & editing), Junpei Yamaguchi (Data curation (clinical), Methodology, Writing—review & editing), Takashi Mizuno (Data curation (clinical), Writing—review & editing), Toshio Kokuryo (Data curation (experimental), Methodology, Writing—review & editing), Hideki Takami, (Data curation (clinical), Investigation, Writing—review & editing), Tomoki Ebata, (Data curation (clinical), Investigation, Writing—review & editing), Tsutomu Fujii, (Supervision, Data curation (clinical), Investigation, Methodology, Writing—review & editing), Yasuhiro Kodera, (Conceptualization, Supervision, Writing—review & editing). All authors approved the final version of the manuscript.

**
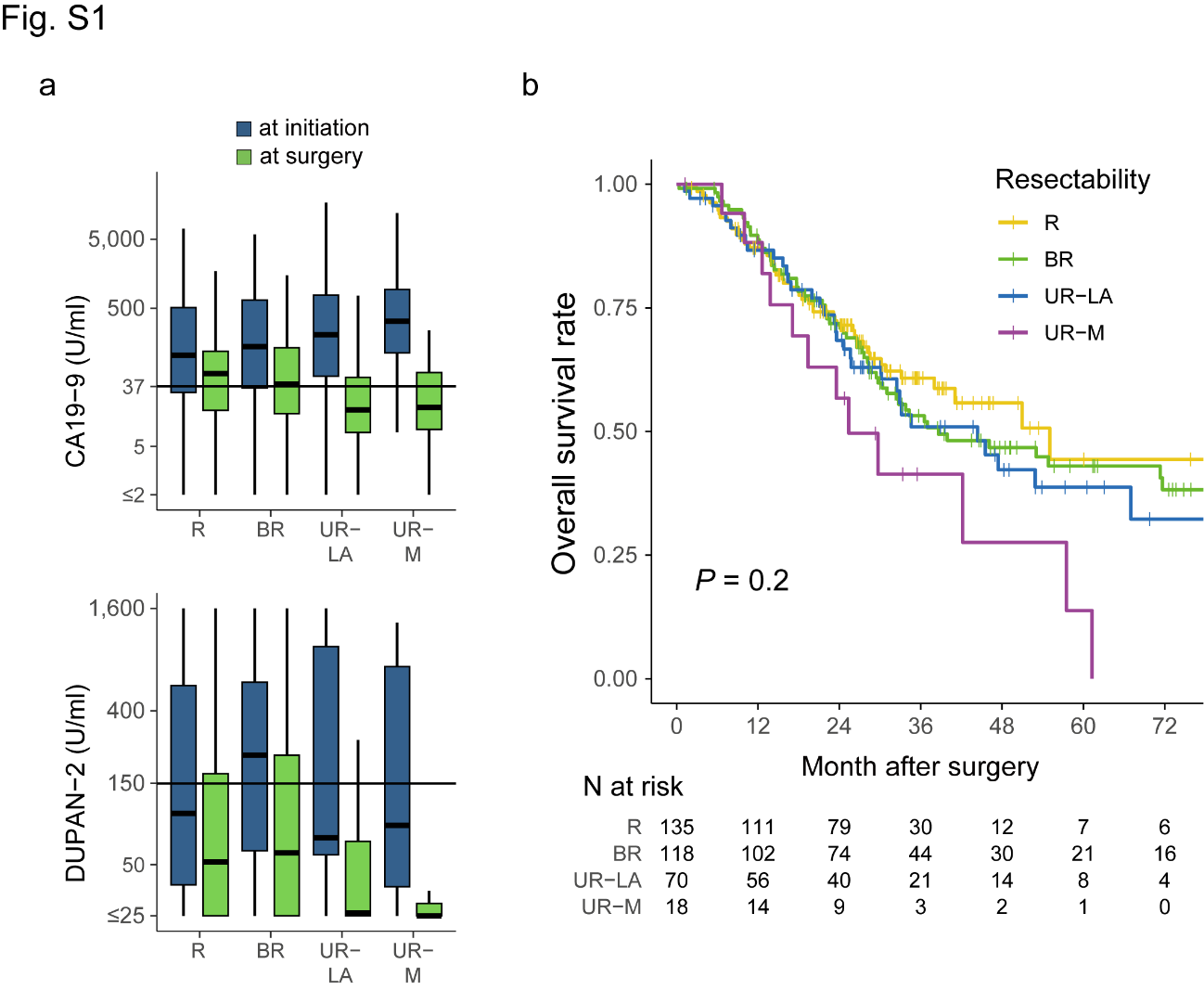
Supplementary Figures and Tables**

**Fig. S1 Sub-analyses in the resectability sub-subset. (a)** Distribution of CA19-9 and DUPAN-2 levels. The y-axis is presented on a log-10 scale. **(b)** Overall survival curve. R, BR, UR-LA, and UR-M indicate resectable, borderline resectable, unresectable due to locally advanced disease, and unresectable due to metastasis, respectively. CA19-9, carbohydrate antigen 19-9.


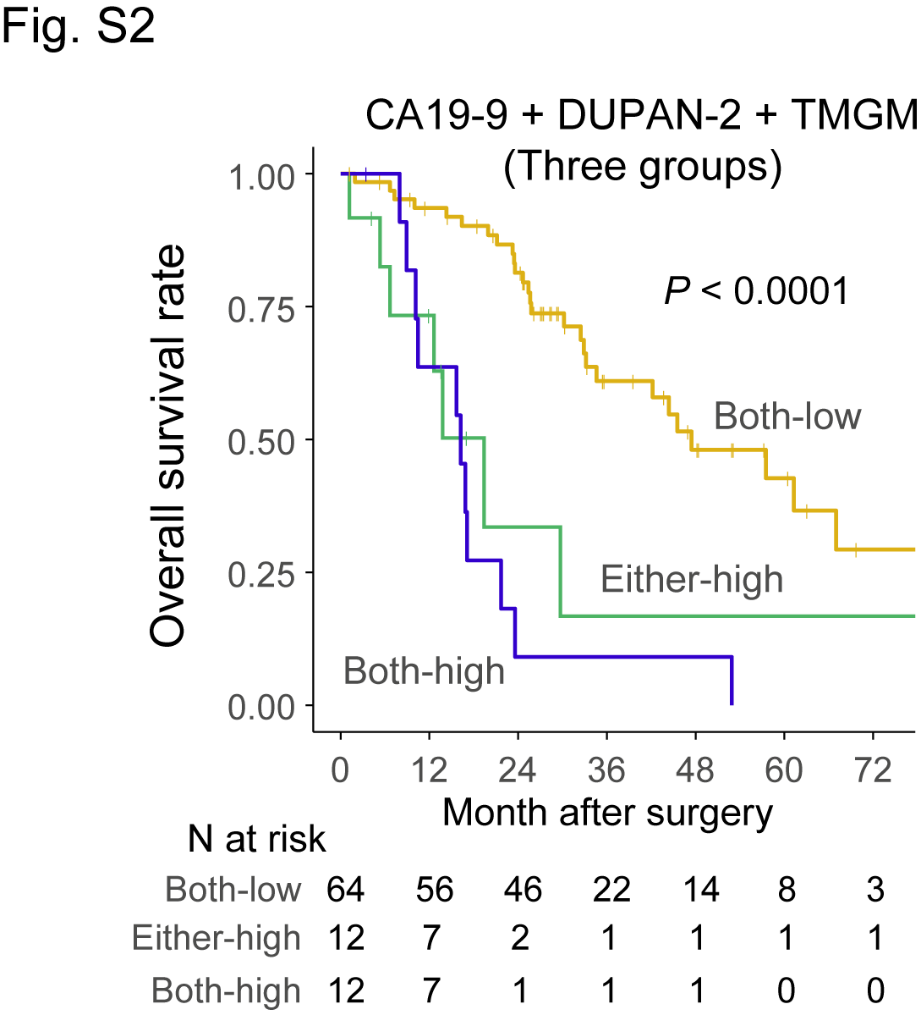


**Fig. S2. Survival curves of the three groups using FUT2/3-specific cutoffs.** Considering these survival curves, the Both-low group was categorized as TMGM-low and the others as TMGM-high (See also **Fig. 1e**). TMGM, tumor gene marker model.

**
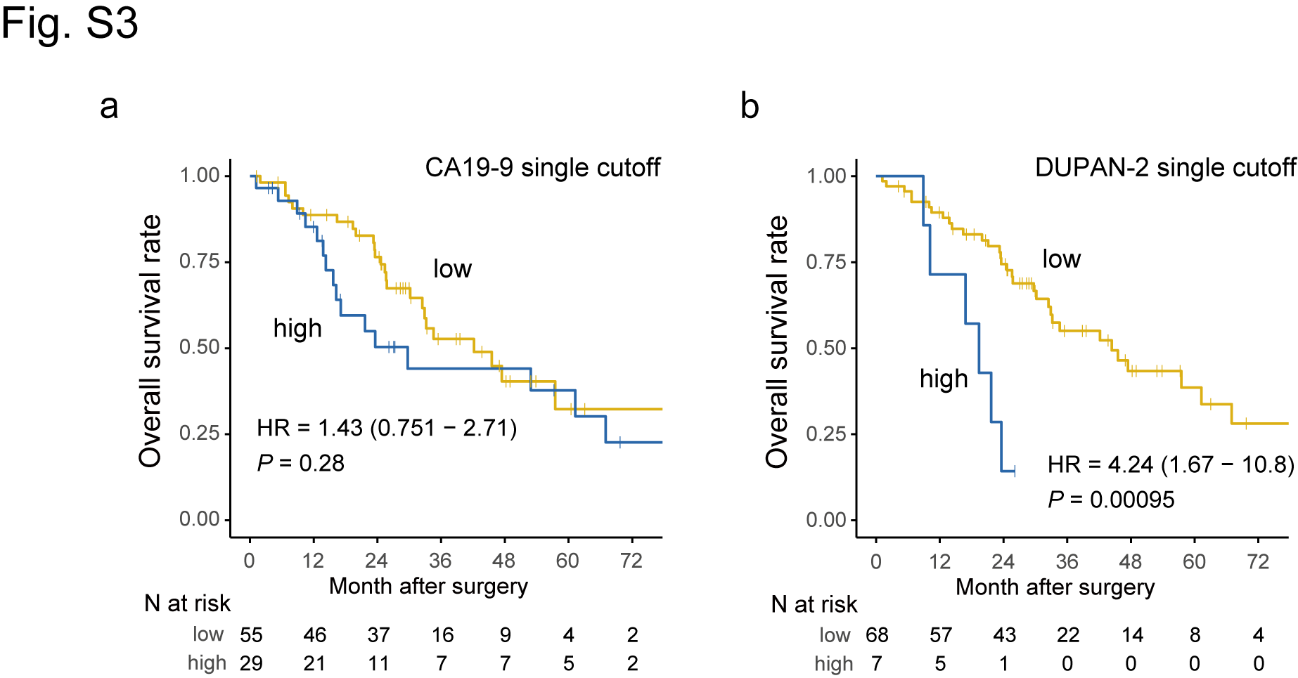
**

**Fig. S3.** **Survival curve analyses using other single-marker cutoff models.** Overall survival curves according to a single-cutoff model using **(a)** CA19-9 and **(b)** DUPAN-2 levels in the unresectable subset. Note that 4 patients whose CA19-9 levels at both initiation of therapy and surgery were ≤ 2 U/ml, and 13 patients with missing DUPAN-2 levels, were not categorized into either high or low groups in these models. CA19-9, carbohydrate antigen 19-9.

**
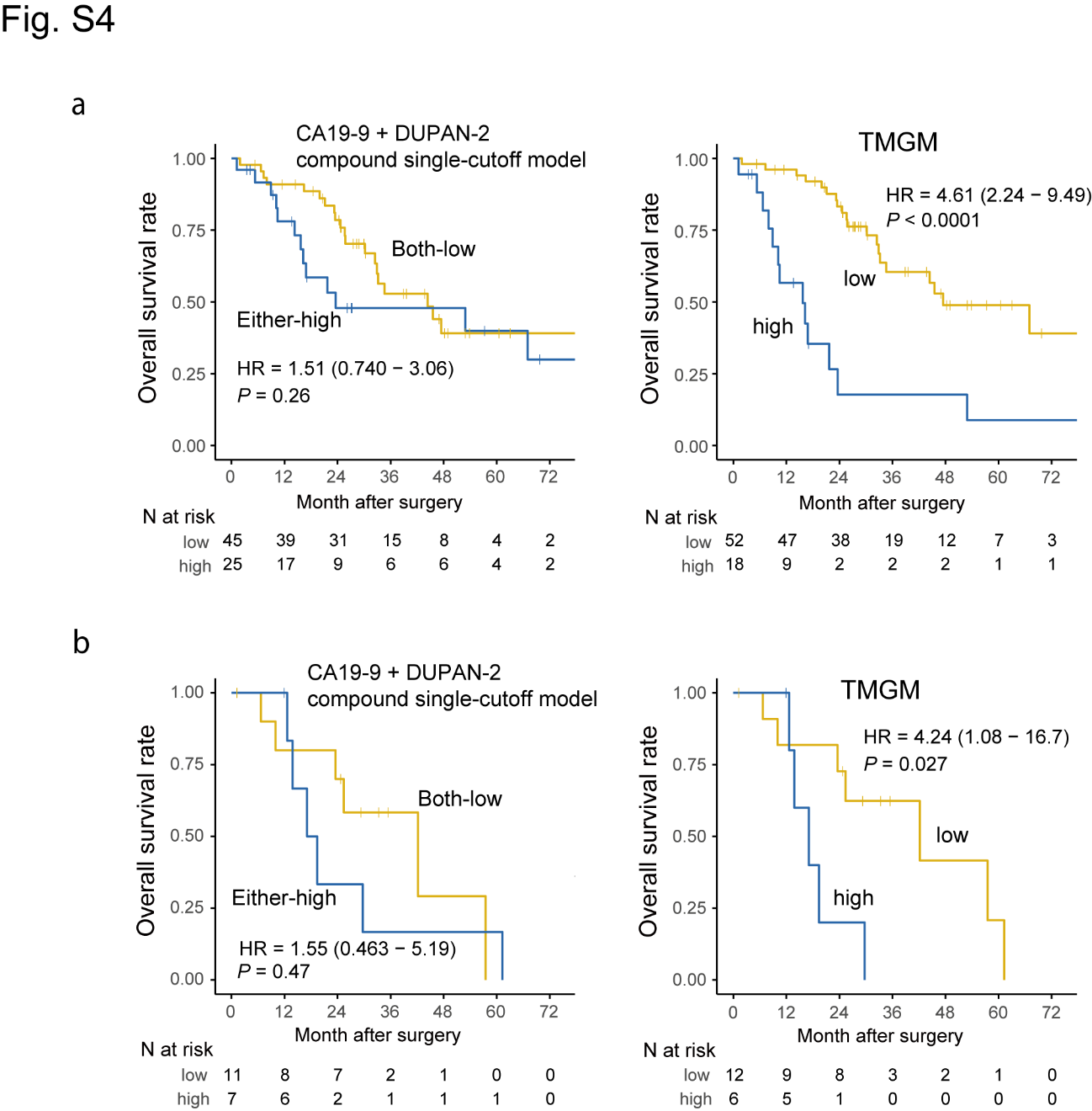
**

**Fig. S4.** **The tumor gene marker model (TMGM) was superior to the compound single-cutoff model regarding stratification of prognoses. Sub-analyses of sub-subsets of unresectable disease.** Overall survival curves according to the compound single-cutoff model (left panel) and TMGM (right panel) in **(a)** locally advanced and **(b)** metastatic disease sub-subsets.

**
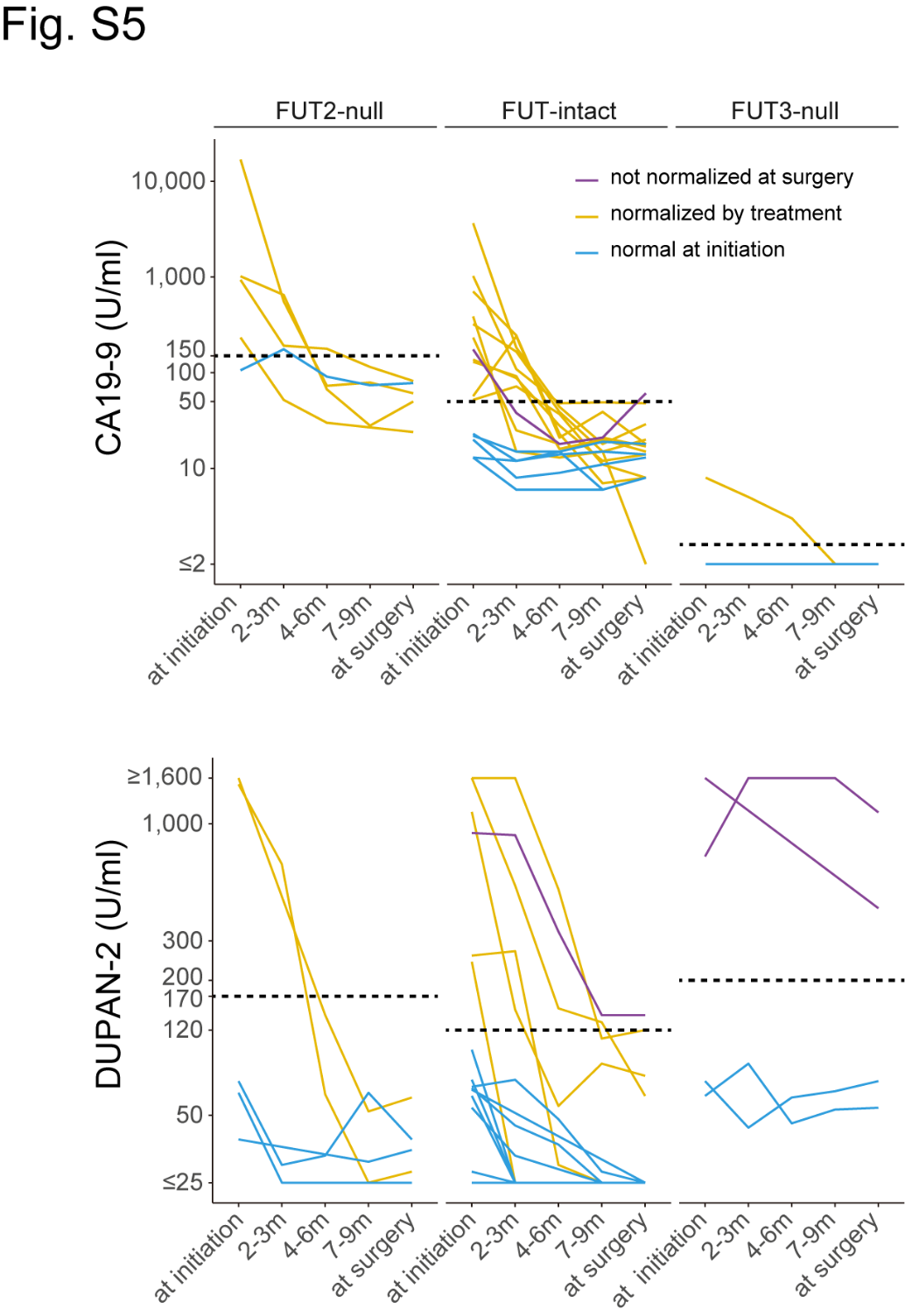
**

**Fig. S5. Chronological tumor marker changes from initiation of induction therapy to conversion surgery stratified by FUT2/3 genotype.** Chronological data were collected for 26 representative patients who underwent preoperative therapy for 8–12 months for unresectable disease. The broken lines indicate the FUT2/3 specific cutoffs. The y-axis is presented on a log-10 scale. CA19-9, carbohydrate antigen 19-9.

| Table S1. List of SNPs characterized in this study | | | | | | |
| --- | --- | --- | --- | --- | --- | --- |
| Assay No. | Gene symbol | variant | dbSNP | Locus (hg19/GRCh37) | variant type | variant allele frequency (information source) |
| 1 | *FUT2* | A385T | rs1047781 | Chr19:49206631 | missense | 0.404 (JGA-SNP),  0.39 (Narimatsu, et al.*) |
| 2 | *FUT2* | C357T | rs281377 | Chr19:49206603 | missense | 0.197 (GEM-WGA), 0.46 (Narimatsu, et al.*) |
| 3 | *FUT3* | T59G | rs28362459 | Chr19:5844792 | missense | 0.305 (JGA-SNP), 0.311 (Narimatsu, et al.*) |
| JGA-SNP, SNP-chip data of the Japanese Genotype-phenotype Archive; GEM-WGA, the GEnome Medical alliance Japan Whole Genome Aggregation Panel. * *Cancer Res* 1998; 58:512–518. (Reference No. 9 in the Supplementary Information) | | | | | | |

| **Table S2. Baseline characteristics of the whole cohort analyzed on the basis of resectability (N=341)** | | | | | |
| --- | --- | --- | --- | --- | --- |
| **Resectability** | **Resectable** | **Borderline resectable** | **Unresectable** | | |
| **n** | **135** | **118** | **88** | **UR-LA** | **UR-M** |
|  |  |  |  | **70** | **18** |
| **Age, years, mean (SD)** | 68.2 (8.3) | 66.8 (8.5) | 66.4 (9.0) | 66.5 (9.1) | 65.9 (8.8) |
| **Sex ratio (M:F)** | 91:44 | 66:52 | 48:40 | 37:33 | 11:7 |
| **Tumor location, n** |  |  |  |  |  |
| head | 19 | 24 | 15 | 13 | 2 |
| body | 84 | 92 | 65 | 55 | 10 |
| tail | 32 | 2 | 8 | 2 | 6 |
| **Tumor diameter, mm** |  |  |  |  |  |
| at diagnosis, mean (SD) | 23.8 (7.3) | 29.8 (16.1) | 33.8 (10.9) | 33.1 (11.1) | 36.4 (10.1) |
| at surgery, mean (SD) | 25.2 (9.3) | 27.0 (10.9) | 22.5 (10.2) | 22.5 (10.7) | 22.2 (8.0) |
| **Initial regimen, n** |  |  |  |  |  |
| GEM/nab-PTX | 27 | 54 | 59 | 48 | 11 |
| FOLFIRINOX | 1 | 30 | 7 | 5 | 2 |
| GEM/S-1 | 90 | 4 | 3 | 3 | 0 |
| GEM | 0 | 0 | 3 | 3 | 0 |
| Others | 17 | 30 | 16 | 11 | 5 |
| **Radiation, n** | 16 | 32 | 38 | 37 | 1 |
| **Treatment duration, months, median [IQR]** | 1.5 [1.1, 2.0] | 2.5 [1.6, 3.7] | 8.2 [5.3, 10.5] | 8.1 [5.0, 10.5] | 8.7 [7.0, 11.8] |
| **RECIST, SD/PR, n** | 125/10 | 98/20 | 41/47 | 34/36 | 7/11 |
| **Surgical procedure, n** |  |  |  |  |  |
| pancreatoduodenectomy | 84 | 89 | 62 | 51 | 11 |
| distal pancreatectomy | 45 | 23 | 22 | 15 | 7 |
| total pancreatectomy | 4 | 6 | 4 | 4 | 0 |
| others | 2 | 0 | 0 | 0 | 0 |
| **Portal vein resection, n** | 38 | 90 | 53 | 45 | 8 |
| **Arterial resection, n** | 3 | 25 | 27 | 25 | 2 |
| **Morbidity, n  (C-D classification)** |  |  |  |  |  |
| None | 47 | 46 | 26 | 20 | 6 |
| I | 9 | 6 | 4 | 4 | 0 |
| II | 32 | 37 | 26 | 21 | 5 |
| IIIa | 45 | 23 | 29 | 22 | 7 |
| IIIb | 1 | 2 | 1 | 1 | 0 |
| IVa | 1 | 3 | 2 | 2 | 0 |
| V (Mortality) | 0 | 1 | 0 | 0 | 0 |
| **Evans classification, n** ¶ |  |  |  |  |  |
| I | 48 | 17 | 5 | 3 | 2 |
| IIa | 56 | 64 | 23 | 16 | 7 |
| IIb | 24 | 26 | 37 | 32 | 5 |
| III | 1 | 4 | 11 | 9 | 2 |
| IV | 1 | 2 | 12 | 10 | 2 |
| **Residual tumor, n** |  |  |  |  |  |
| R0 | 119 | 88 | 70 | 58 | 12 |
| R1 | 15 | 28 | 16 | 11 | 5 |
| R2 | 0 | 2 | 2 | 1 | 1 |
| X | 1 | 0 | 0 | 0 | 0 |
| ¶ missing information in 10 cases; UR-LA indicates unresectable for locally advanced lesions; UR-M unresectable for metastatic disease; C–D, Clavien–Dindo; SD, standard deviation; IQR, interquartile range; GEM/nab-PTX, gemcitabine | | | | | |

| Table S3. Characteristics of the patients with metastatic disease | |
| --- | --- |
| Variables | **n=18** |
| Resectability of primary disease |  |
| Resectable | 14 |
| Borderline resectable | 1 |
| Locally advanced | 3 |
| Organ of metastasis |  |
| liver | 7 |
| peritoneum | 5 |
| distant lymph node | 3 |
| peritoneal cytology | 2 |
| liver and peritoneum | 1 |
| (Cyto)pathological proof of metastasis | 11 |
| Number of macroscopic metastatic foci |  |
| 1 | 5 |
| 2 | 1 |
| 3 | 1 |
| 4 or more | 9 |
| not applicable (peritoneal cytology) | 2 |
| Presence of target lesion during induction therapy * | 11 |
| Resection of metastases at conversion surgery |  |
| sampling | 9 |
| resected at the initial assessment | 1 |
| total resection | 4 |
| unable to identify | 4 |
| * Response Evaluation Criteria in Solid Tumors (version 1.1) | |

| **Table S4. Baseline characteristics of the patients with unresectable disease** | | | | |
| --- | --- | --- | --- | --- |
| **FUT2/3 genotype** | **FUT2-null** | **FUT-intact** | **FUT3-null** | ***P*** |
| **n** | **9** | **73** | **6** |  |
| **Metastatic disease, n** | 1 | 16 | 1 | 0.73 |
| **Age, years, mean (SD)** | 64.7 (13.9) | 66.7 (8.4) | 64.8 (9.2) | 0.75 |
| **Sex ratio (M:F)** | 4:5 | 41:32 | 3:3 | 0.78 |
| **Tumor location, n** |  |  |  | 0.64 |
| head | 0 | 14 | 1 |  |
| body | 8 | 53 | 4 |  |
| tail | 1 | 6 | 1 |  |
| **Tumor diameter, mm** |  |  |  |  |
| at diagnosis, mean (SD) | 31.0 (9.1) | 34.2 (11.3) | 33.2 (9.4) | 0.71 |
| at surgery, mean (SD) | 20.5 (7.6) | 22.4 (10.7) | 25.5 (7.1) | 0.66 |
| **Initial regimen, n** |  |  |  | 0.49 |
| GEM/nab-PTX | 7 | 49 | 3 |  |
| FOLFIRINOX | 1 | 4 | 2 |  |
| GEM/S-1 | 0 | 3 | 0 |  |
| GEM | 0 | 3 | 0 |  |
| Others | 1 | 14 | 1 |  |
| **Radiation, n** | 4 | 30 | 4 | 0.48 |
| **Treatment duration, month, median [IQR]** | 9.8 [8.2, 12.0] | 8.0 [4.8, 10.3] | 10.0 [7.8, 11.6] | 0.16 |
| **RECIST, SD/PR, n** | 4/5 | 32/41 | 5/1 | 0.17 |
| **Surgical procedure, n** |  |  |  | 0.70 |
| pancreatoduodenectomy | 8 | 50 | 4 |  |
| distal pancreatectomy | 1 | 19 | 2 |  |
| total pancreatectomy | 0 | 4 | 0 |  |
| **Portal vein resection, n** | 4 | 45 | 4 | 0.58 |
| **Arterial resection, n** | 1 | 25 | 1 | 0.27 |
| **Morbidity, n (C–D classification)** |  |  |  | 0.97 |
| None | 1 | 23 | 2 |  |
| I | 1 | 3 | 0 |  |
| II | 3 | 21 | 2 |  |
| IIIa | 4 | 23 | 2 |  |
| IIIb | 0 | 1 | 0 |  |
| IVa | 0 | 2 | 0 |  |
| **Evans classification, n** |  |  |  | 0.30 |
| I | 0 | 5 | 0 |  |
| IIa | 5 | 17 | 1 |  |
| IIb | 2 | 30 | 5 |  |
| III | 1 | 10 | 0 |  |
| IV | 1 | 11 | 0 |  |
| **Residual tumor, n** |  |  |  | 0.55 |
| 0 | 6 | 60 | 4 |  |
| 1 | 3 | 11 | 2 |  |
| 2 | 0 | 2 | 0 |  |

SD, standard deviation; GEM/nab-PTX, gemcitabine plus nab-paclitaxel; GEM, gemcitabine; IQR, interquartile range; RECIST, Response Evaluation Criteria in Solid Tumors version 1.1; SD/PR, stable disease/partial response; C–D, Clavien–Dindo.

**References**

1. Unno M, Motoi F, Matsuyama Y, Matsuyama Y. Randomized phase II/III trial of neoadjuvant chemotherapy with gemcitabine and S-1 versus upfront surgery for resectable pancreatic cancer (Prep-02/JSAP-05). ***J Clin Oncol.*** 2019;37(Suppl 4):189.

2. Yamaguchi J, Yokoyama Y, Fujii T, Yamada S, Takami H, Kawashima H, et al. Results of a Phase II Study on the Use of Neoadjuvant Chemotherapy (FOLFIRINOX or GEM/nab-PTX) for Borderline-resectable Pancreatic Cancer (NUPAT-01). ***Ann Surg.*** 2022;275:1043–1049.

3. Igarashi T, Yamada S, Hoshino Y, Murotani K, Baba H, Takami H, et al. Prognostic factors in conversion surgery following nab-paclitaxel with gemcitabine and subsequent chemoradiotherapy for unresectable locally advanced pancreatic cancer: Results of a dual-center study. ***Ann Gastroenterol Surg.*** 2023;7:157–166.

4. Igarashi T, Fukasawa M, Watanabe T, Kimura N, Itoh A, Tanaka H, et al. Evaluating staging laparoscopy indications for pancreatic cancer based on resectability classification and treatment strategies for patients with positive peritoneal washing cytology. ***Ann Gastroenterol Surg.*** 2024;8:124–132.

5. Hashimoto D, Satoi S, Fujii T, Sho M, He J, Hackert T, et al. Is surgical resection justified for pancreatic ductal adenocarcinoma with distant abdominal organ metastasis? A position paper by experts in pancreatic surgery at the Joint Meeting of the International Association of Pancreatology (IAP) & the Japan Pancreas Society (JPS) 2022 in Kyoto. ***Pancreatology.*** 2023;23:682–688.

6. Omiya K, Maekawa A, Oba A, Inoue Y, Hirose Y, Kobayashi K, et al. A proposal of ABCD metastasectomy criteria for synchronous/metachronous metastatic pancreatic cancer in the era of multidisciplinary treatment. ***Br J Surg.*** 2024;111.

7. Tanaka H, Tamura K, Abe T, Yoshida T, Macgregor-Das A, Dbouk M, et al. Serum Carboxypeptidase Activity and Genotype-Stratified CA19-9 to Detect Early-Stage Pancreatic Cancer. ***Clin Gastroenterol Hepatol.*** 2022;20:2267–2275 e2262.

8. Tanaka H, Kanda M, Shimizu D, Tanaka C, Inokawa Y, Hattori N, et al. Transcriptomic profiling on localized gastric cancer identified CPLX1 as a gene promoting malignant phenotype of gastric cancer and a predictor of recurrence after surgery and subsequent chemotherapy. ***J Gastroenterol.*** 2022;57:640–653.

9. Narimatsu H, Iwasaki H, Nakayama F, Ikehara Y, Kudo T, Nishihara S, et al. Lewis and secretor gene dosages affect CA19-9 and DU-PAN-2 serum levels in normal individuals and colorectal cancer patients. ***Cancer Res.*** 1998;58:512–518.

10. Abe T, Koi C, Kohi S, Song KB, Tamura K, Macgregor-Das A, et al. Gene Variants That Affect Levels of Circulating Tumor Markers Increase Identification of Patients With Pancreatic Cancer. ***Clin Gastroenterol Hepatol.*** 2020;18:1161–1169.e1165.

11. Ando Y, Dbouk M, Yoshida T, Saba H, Abou Diwan E, Yoshida K, et al. Using tumor marker gene variants to improve the diagnostic accuracy of DUPAN-2 and carbohydrate antigen 19-9 for pancreatic cancer. ***J Clin Oncol.*** 2024:JCO2301573.

12. Harrell FE, Jr., Califf RM, Pryor DB, Lee KL, Rosati RA. Evaluating the yield of medical tests. ***JAMA.*** 1982;247:2543–2546.

13. Chen Y, Huang J, He X, Gao Y, Mahara G, Lin Z, et al. A novel approach to determine two optimal cut-points of a continuous predictor with a U-shaped relationship to hazard ratio in survival data: simulation and application. ***BMC Med Res Methodol.*** 2019;19:96.

14. Posta M, Gyorffy B. Analysis of a large cohort of pancreatic cancer transcriptomic profiles to reveal the strongest prognostic factors. ***Clin Transl Sci.*** 2023;16:1479–1491.
